# Possible risk factors for tip rupture of orbital atherectomy system

**DOI:** 10.1007/s12928-021-00768-5

**Published:** 2021-03-05

**Authors:** Hiromasa Katoh, Yoshihiro Noji, Masato Yamaguchi

**Affiliations:** Department of Cardiology, Cardiovascular Center, Fukui Prefectural Hospital, 2-8-1 Yotsui, Fukui City, Fukui 910-8526 Japan

**Keywords:** Calcified lesion, Orbital atherectomy system, Tip rupture

Orbital atherectomy system (OAS) is effective for treatment of severely calcified coronary lesions [1]. On the other hand, tip rupture of OAS sometimes relates to critical vessel injury such as vessel rupture or dissection. Thus, we have to consider risk factors of this phenomenon. We examined consecutive four cases (9.5%, 4/42 lesions) which occurred OAS-tip rupture. All cases had acute bend (a cut-off value of ≤ 120.8 degree was calculated by receiver operating characteristics curve analysis, Supplement data 1 to 4) and nodular calcification at the target lesion (Fig. 1a, c, e, h). Debulking with OAS was performed at 80,000 rpm with backward advancement in three cases and forward advancement in one case (Case 2). Tip rupture was occurred within ten times OAS activation in all cases and ruptured tip was successfully and easily retrieved using the illustrated method in Case 1 to 3 (Fig. 2; Supplement Movie 1 and 2). All ruptured sites were observed at just proximal portion of the crown (Fig. 1g). In case 4, we advanced the crown beyond the target lesion with GlideAssist mode and started sanding at 80,000 rpm with backward advancement. Because we detected a radiolucent band just proximal portion of the crown (Pre-rupture sign) during an initial activation (Fig. 1i), we removed the OAS carefully and identified the prolonged OAS shaft (Fig. 1j). Vessel tortuosity and nodular calcification may lead to unevenness of rotation between crown and proximal shaft (Fig. 1k). For safety procedure with OAS, it is important to be aware of these risk factors and pre-rupture sign.

**Fig. 1:**
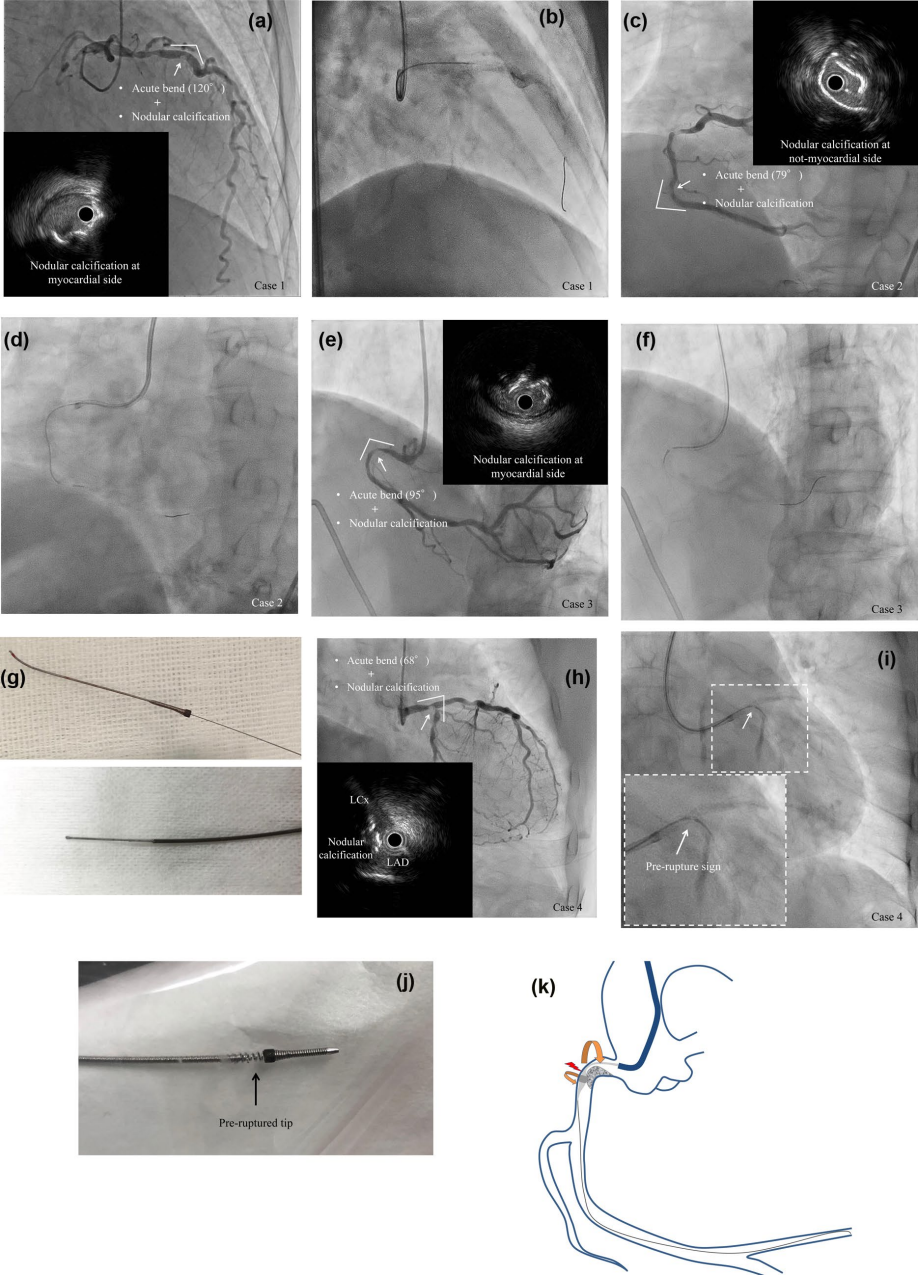
OAS tip rupture cases and a speculation of this phenomenon. **a** Initial angiogram and IVUS image of case 1 (white arrow points to nodular calcification). **b** Rupture of OAS tip (Case 1). **c** Initial angiogram and IVUS image of case 2. **d** Rupture of OAS tip (Case 2). **e** Initial angiogram and IVUS image of case 3. **f** Rupture of OAS tip (Case 3). **g** Ruptured tip of OAS and its shaft. h. Initial angiogram and IVUS image of case 4. **i** Radiolucent band reveals pre-ruptured tip of OAS. **j** Pre-rupture of OAS tip (Case 4). **k** Speculation over causes of OAS tip rupture. IVUS, intravascular ultrasound; OAS, orbital atherectomy system

**Fig. 2:**
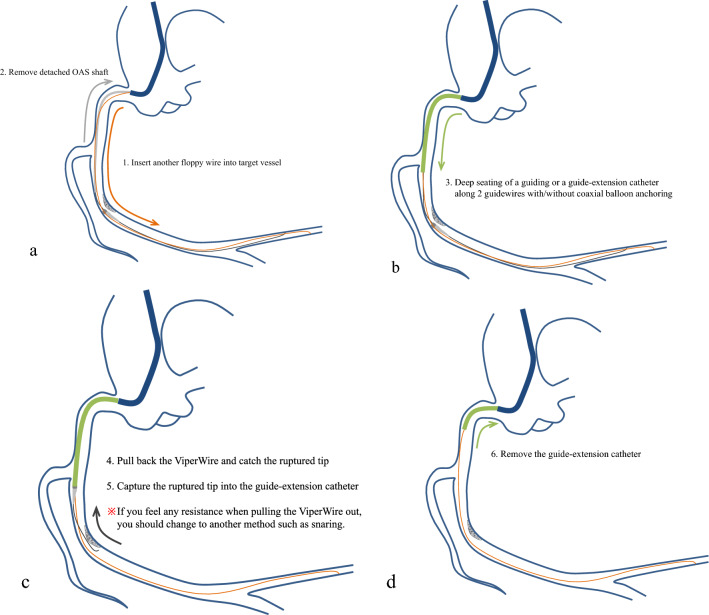
How to retrieve the ruptured tip. Step 1. Insert another floppy guidewire into the target vessel. Step 2. Remove detached OAS shaft. Step 3. Deep seating of a guiding or a guide-extension catheter along two guidewires with or without coaxial balloon anchoring technique. Step 4. Pull back the ViperWire Advance Flex Tip (Medikit, Tokyo, Japan) and catch the ruptured tip. Step 5. Capture the ruptured tip into the guide-extension or the guiding catheter. ※If you feel any resistance when pulling the ViperWire out, you should change to another method such as snaring

## Supplementary Information

Below is the link to the electronic supplementary material.

**Supplementary file 1: Data 4.** Receiver operating characteristics curve analysis. At a cut-off value of 120.8 degree, angle of bend exhibited 100% sensitivity and 89.5 specificity for predicting tip rupture of orbital atherectomy system (PPTX 87 KB).

**Supplementary file 2: Movie 1.** Retrieval of the ruptured tip in Case 2. At first, we inserted another floppy guidewire into the target vessel. As the second step, we removed the detached shaft of OAS. As the third step, we inserted the guide-extension catheter into the target vessel deeply along two guidewires. As the fourth step, we pulled back the ViperWire and caught the ruptured tip of OAS. Finally, we could successfully capture the ruptured tip into the guide-extension catheter and retrieve it. OAS, orbital atherectomy system (MPG 15206 KB).

**Supplementary file 3: Movie 2.** Retrieval of the ruptured tip in Case 3. At first, we inserted another floppy guidewire into the target vessel. As the second step, we removed the detached shaft of OAS. As the third step, we engaged the guiding catheter into the right coronary artery deeply and coaxially. Finally we pulled back the ViperWire, caught the ruptured tip of OAS, and captured the ruptured tip into the guiding catheter. OAS, orbital atherectomy system (MPG 8948 KB).
