# Supplementary material for: Possible risk factors for tip rupture of orbital atherectomy system
Source: Cardiovasc Interv Ther. 2021 Mar 5;37(2):385–8. doi: 10.1007/s12928-021-00768-5 (PMC8926987; doi:10.1007/s12928-021-00768-5)
Supplement: Supplementary file 1 — Data 4. Receiver operating characteristics curve analysis. At a cut-off value of 120.8 degree, angle of bend exhibited 100% sensitivity and 89.5 specificity for predicting tip rupture of orbital atherectomy system (PPTX 87 KB). [file 12928_2021_768_MOESM1_ESM.pptx]

## Slide 1
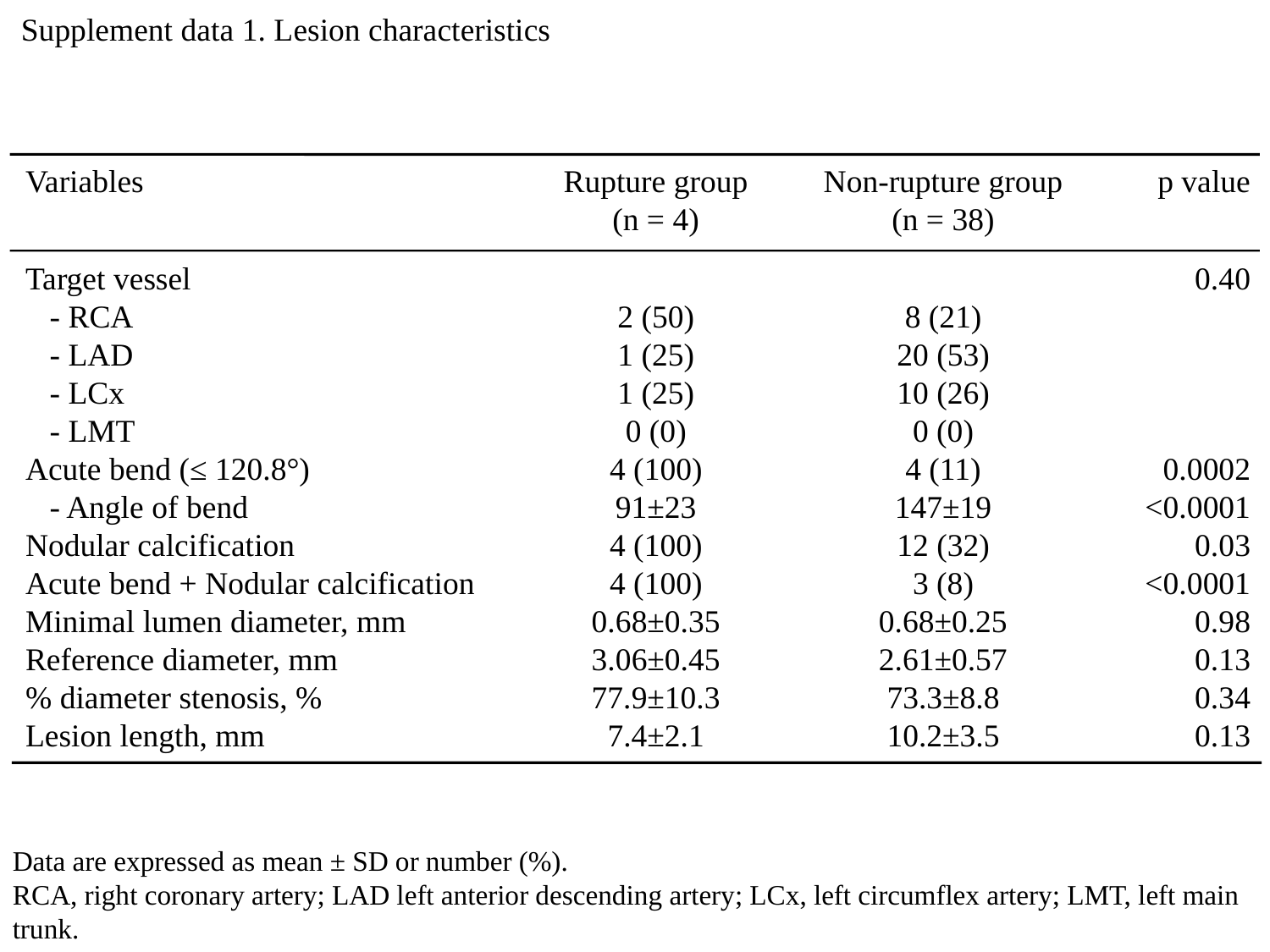

Supplement data 1. Lesion characteristics
Variables
Target vessel
 - RCA
 - LAD
 - LCx
 - LMT
Acute bend (≤ 120.8°)
 - Angle of bend
Nodular calcification
Acute bend + Nodular calcification
Minimal lumen diameter, mm
Reference diameter, mm
% diameter stenosis, %
Lesion length, mm
Rupture group
(n = 4)
2 (50)
1 (25)
1 (25)
0 (0)
4 (100)
91±23
4 (100)
4 (100)
0.68±0.35
3.06±0.45
77.9±10.3
7.4±2.1
Non-rupture group
(n = 38)
8 (21)
20 (53)
10 (26)
0 (0)
4 (11)
147±19
12 (32)
3 (8)
0.68±0.25
2.61±0.57
73.3±8.8
10.2±3.5
p value
0.40
0.0002
<0.0001
0.03
<0.0001
0.98
0.13
0.34
0.13
Data are expressed as mean ± SD or number (%).
RCA, right coronary artery; LAD left anterior descending artery; LCx, left circumflex artery; LMT, left main trunk.

## Slide 2
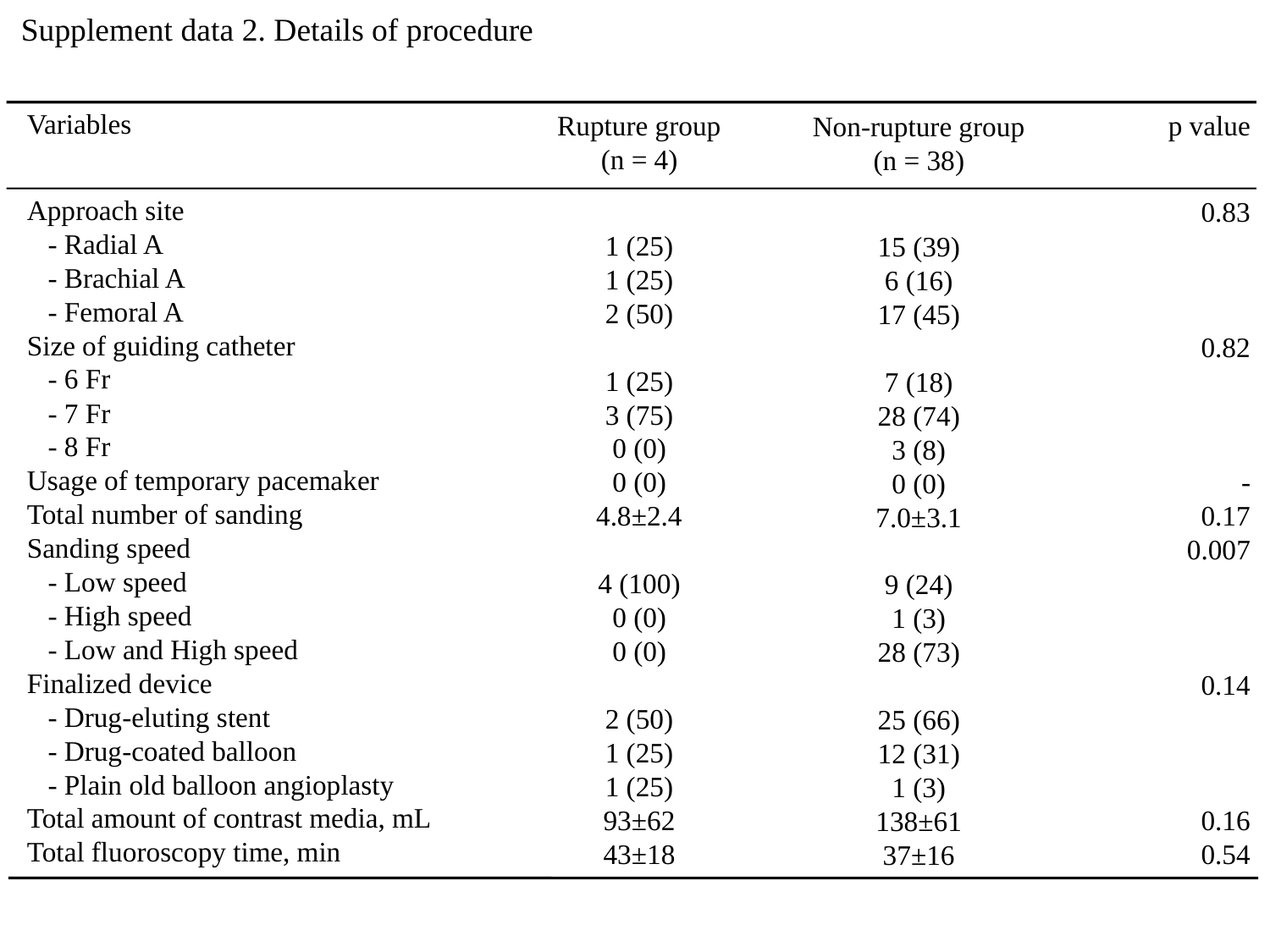

Supplement data 2. Details of procedure
Variables
Approach site
 - Radial A
 - Brachial A
 - Femoral A
Size of guiding catheter
 - 6 Fr
 - 7 Fr
 - 8 Fr
Usage of temporary pacemaker
Total number of sanding
Sanding speed
 - Low speed
 - High speed
 - Low and High speed
Finalized device
 - Drug-eluting stent
 - Drug-coated balloon
 - Plain old balloon angioplasty
Total amount of contrast media, mL
Total fluoroscopy time, min
Rupture group
(n = 4)
1 (25)
1 (25)
2 (50)
1 (25)
3 (75)
0 (0)
0 (0)
4.8±2.4
4 (100)
0 (0)
0 (0)
2 (50)
1 (25)
1 (25)
93±62
43±18
p value
0.83
0.82
-
0.17
0.007
0.14
0.16
0.54
Non-rupture group
(n = 38)
15 (39)
6 (16)
17 (45)
7 (18)
28 (74)
3 (8)
0 (0)
7.0±3.1
9 (24)
1 (3)
28 (73)
25 (66)
12 (31)
1 (3)
138±61
37±16

## Slide 3
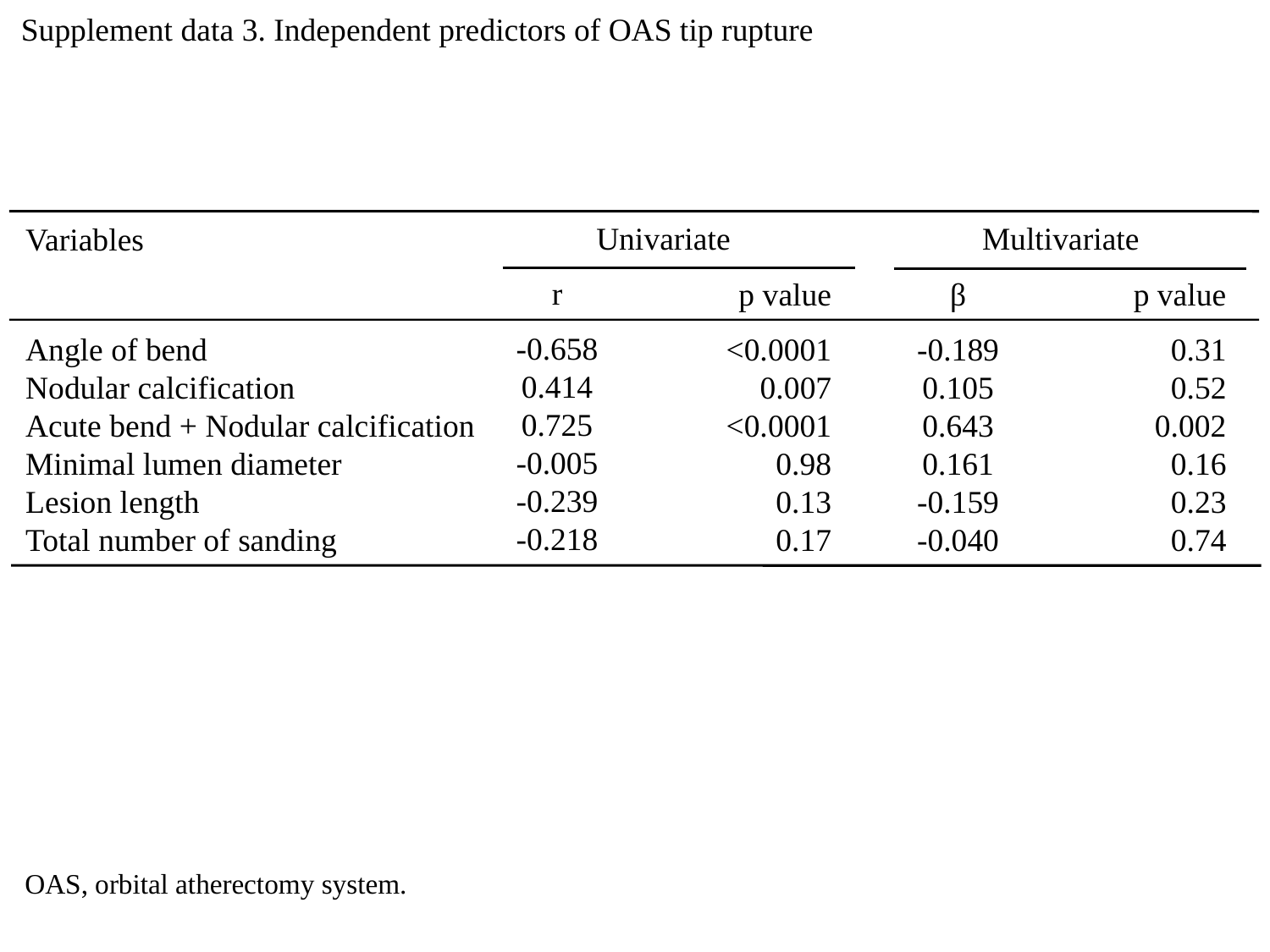

Supplement data 3. Independent predictors of OAS tip rupture
Univariate
r
-0.658
0.414
0.725
-0.005
-0.239
-0.218
Multivariate
Variables
Angle of bend
Nodular calcification
Acute bend + Nodular calcification
Minimal lumen diameter
Lesion length
Total number of sanding
p value
<0.0001
0.007
<0.0001
0.98
0.13
0.17
β
-0.189
0.105
0.643
0.161
-0.159
-0.040
p value
0.31
0.52
0.002
0.16
0.23
0.74
OAS, orbital atherectomy system.

## Slide 4
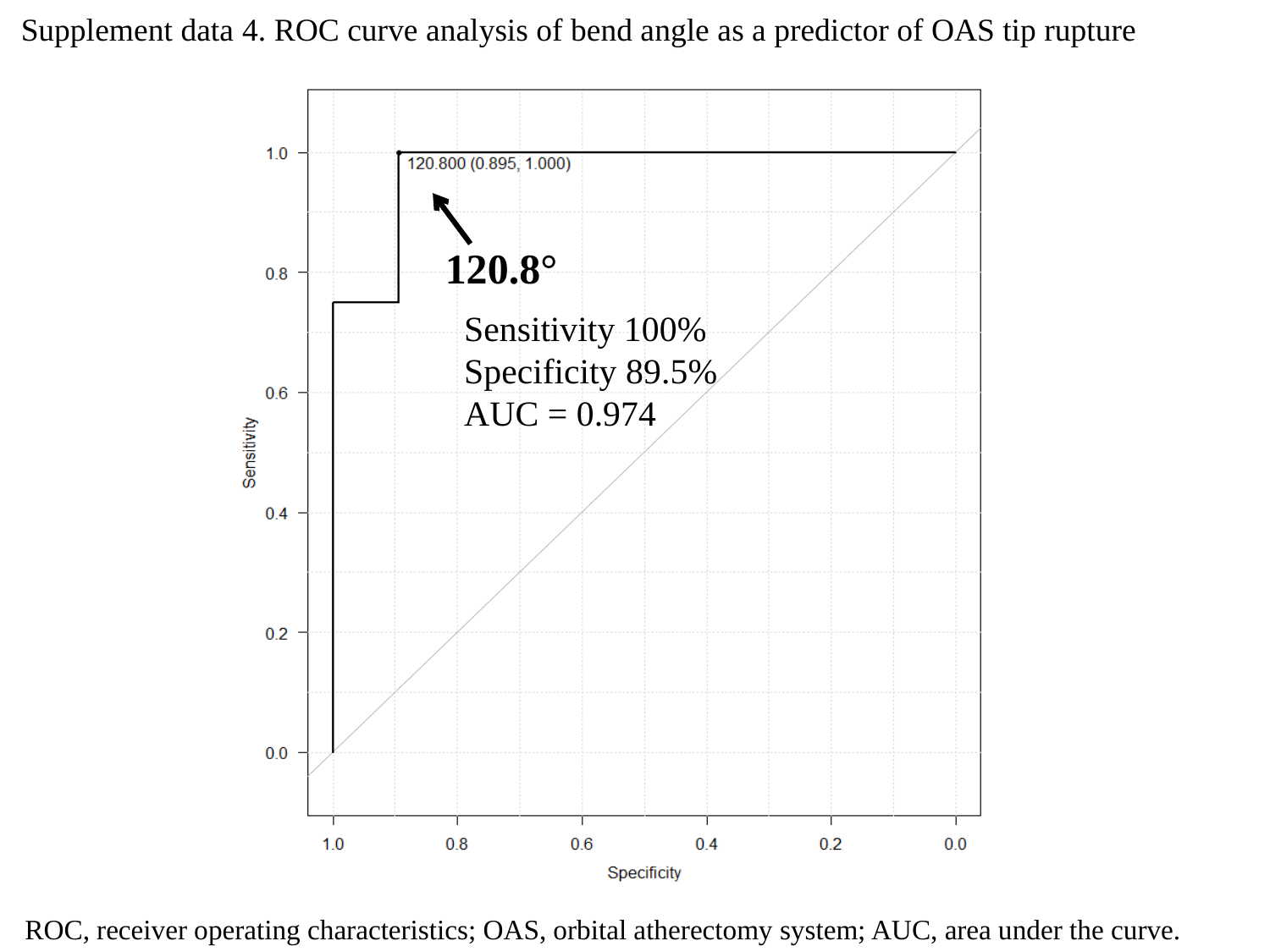

Supplement data 4. ROC curve analysis of bend angle as a predictor of OAS tip rupture
120.8°
Sensitivity 100%
Specificity 89.5%
AUC = 0.974
ROC, receiver operating characteristics; OAS, orbital atherectomy system; AUC, area under the curve.
